# Supplementary material for: Projecting the long-term effects of the COVID-19 pandemic on U.S. population structure
Source: Nat Commun. 2024 Mar 18;15:2409. doi: 10.1038/s41467-024-46582-4 (PMC10948855; doi:10.1038/s41467-024-46582-4)
Supplement: Supplementary file 1 — Supplementary Information [file 41467_2024_46582_MOESM1_ESM.pdf]

"Projecting the Long-term Effects of the COVID-19 Pandemic on U.S. Population Structure"
Appendix

I. Supplementary Methods
   a. Comparison of UNWPP input data and US final estimates
   b. Comparison of different migration estimates
   c. Comparison of projections from UNWPP, Census IDB, CBO
   d. All manuscript graphs, ending at 2040
   e. Projected change in population size and summary table
   f. Cohort component projection method
   g. Data sources for supplementary analyses

I. Supplementary Methods

A. Comparison of UNWPP input data and US final estimates

Here we compare the UNWPP input data with several data sources. For mortality, we compare with (1) Human Mortality Database (HMD) and (2) National Vital Statistics System (NVSS) (Appendix Figures 1 and 2). For 2019 and 2020, mortality rates (m_x) and probabilities of dying (q_x) are nearly identical between sources for ages 0-85. HMD provides estimates to age 110+, while UNWPP and NVSS final life tables truncate at 100+, hence the discrepancies at the last 10 years. NVSS has not yet released their final mortality estimates for 2021, but rates/probabilities for 2019 and 2020 are close to those used by UNWPP.

Supplementary Figure 1: Mortality rate (m_x) comparison, shown on log scale

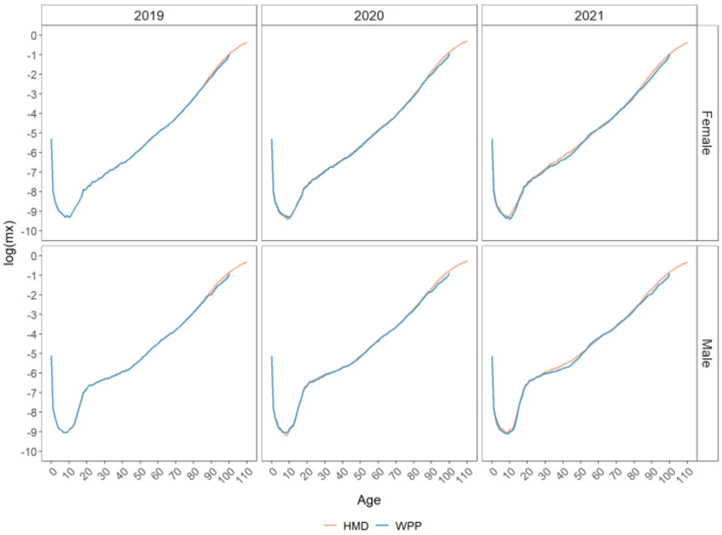

Supplementary Figure 2: Probability of dying (q_x) comparison

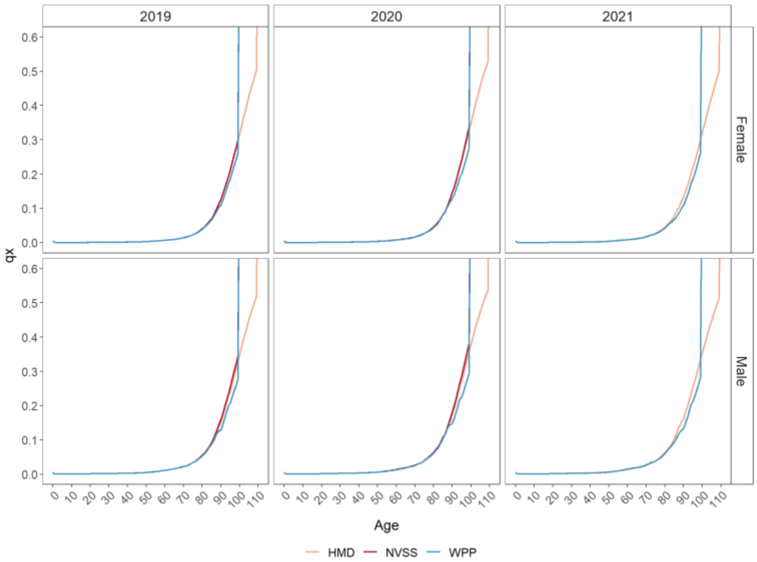

For fertility, we compare with (1) Human Fertility Database (HFD) (Appendix Figure 3) and (2) National Vital Statistics System (NVSS) (Appendix Figure 4). Final birth data

from NVSS are only available in five-year age groups, but those final estimates from NVSS are incredibly close to the estimates used by UNWPP.

Supplementary Figure 3: ASFR comparison (single ages)

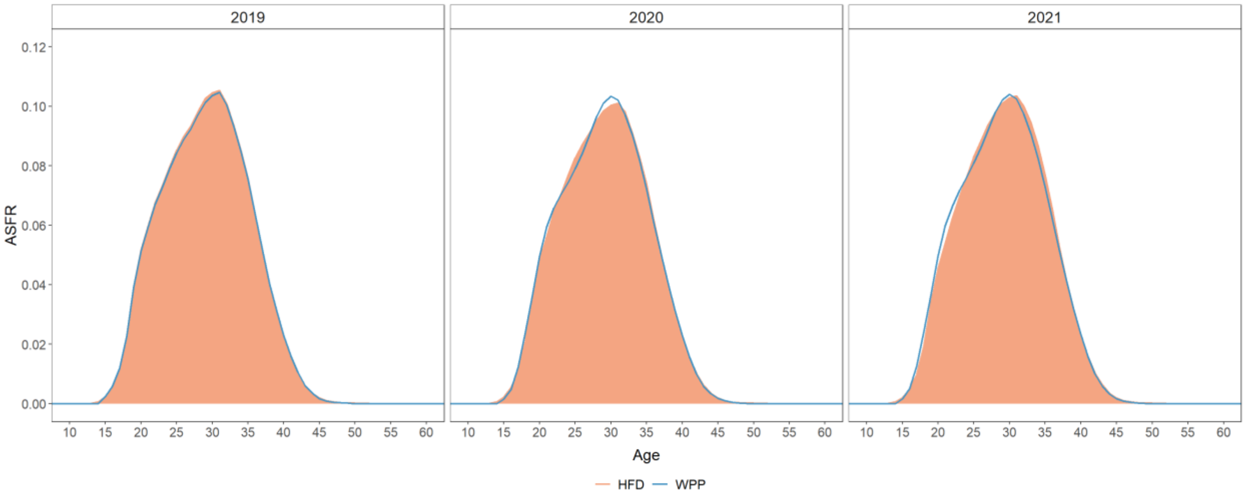

Supplementary Figure 4: ASFR comparison (five-year age groups)

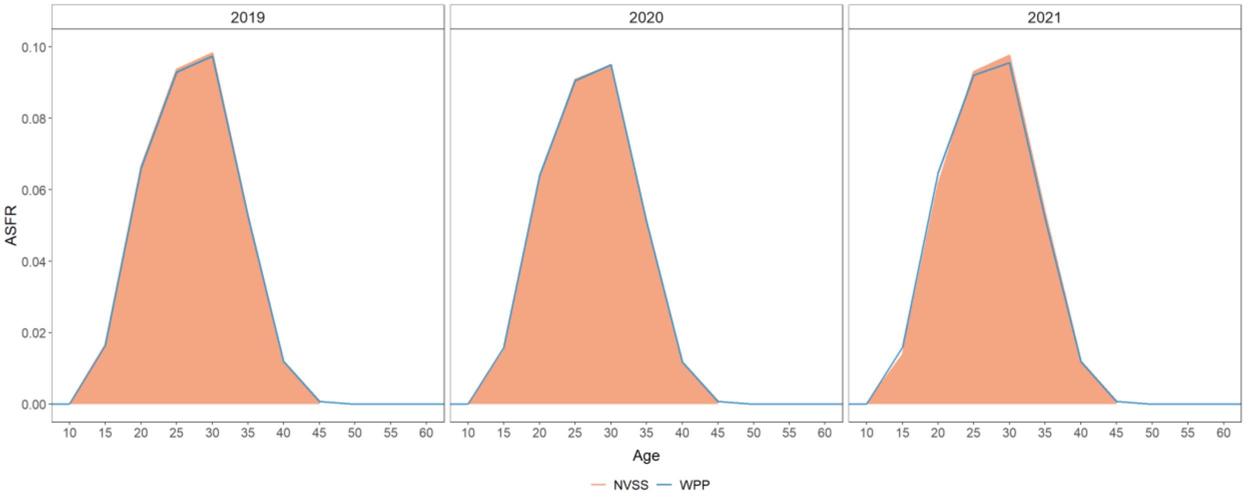

B. Comparison of different migration estimates

Now we compare the validity of the migration estimates from the UNWPP to those from the US Census Bureau. The UNWPP uses the data from the US Census Bureau as input for their final estimates. Although the notable difference is that the Census Bureau reports these values in fiscal year (July 1 in Year 1 to June 30 in Year 2), while the UNWPP uses calendar year.

Thus, we take the fiscal year migration estimates from Census Bureau for 2019, 2020, 2021, and 2022, convert them into calendar year, and substitute these in for the values for the migration estimates from the UNWPP for the years 2020 and 2021 in our baseline scenario.

We present two figures to compare these. Appendix Figure 5 is a replication of Figure 2 A from the main text, but with the migration values replaced for 2020 and 2021. Appendix Figure 6 is an exact replication of Figure 2 A from the main text. These figures are nearly identical in shape.

Supplementary Figure 5: Absolute Difference in Population Size by Age and Sex for Years 2025 (left), 2040 (middle), 2060 (right). Migration Estimates from Census Bureau for 2020 and 2021

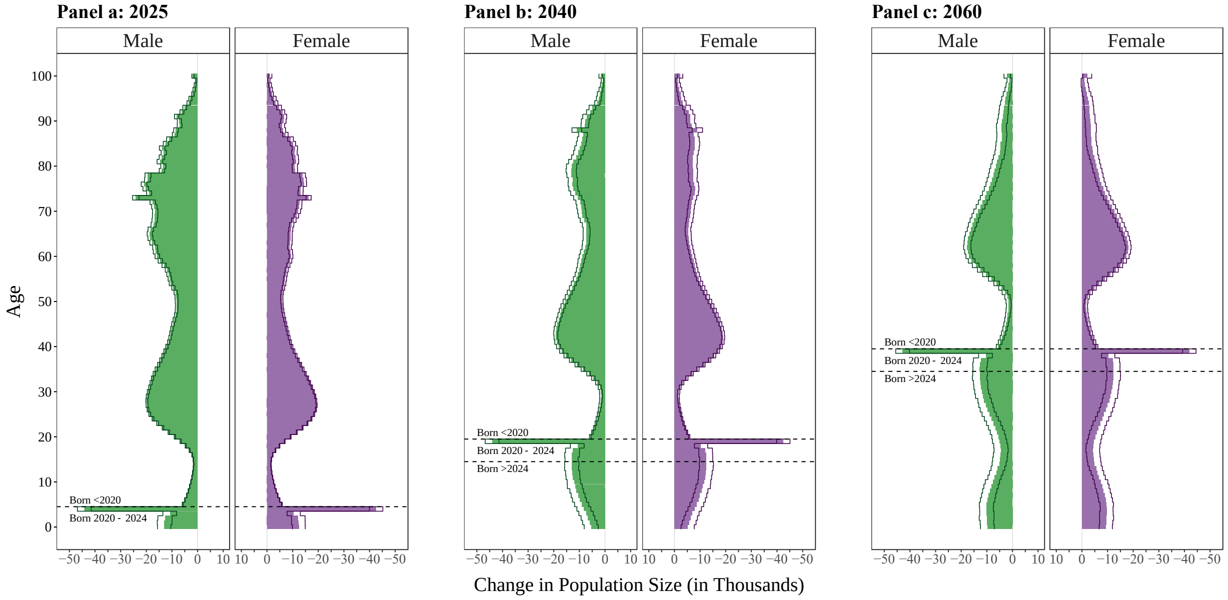

Dashed lines represent 95% confidence intervals.

Supplementary Figure 6: Absolute Difference in Population Size by Age and Sex for Years 2025 (left), 2040 (middle), 2060 (right). Migration Estimates from WPP for 2020 and 2021 (Note: this is the same as Figure 2, Panel A in main manuscript)

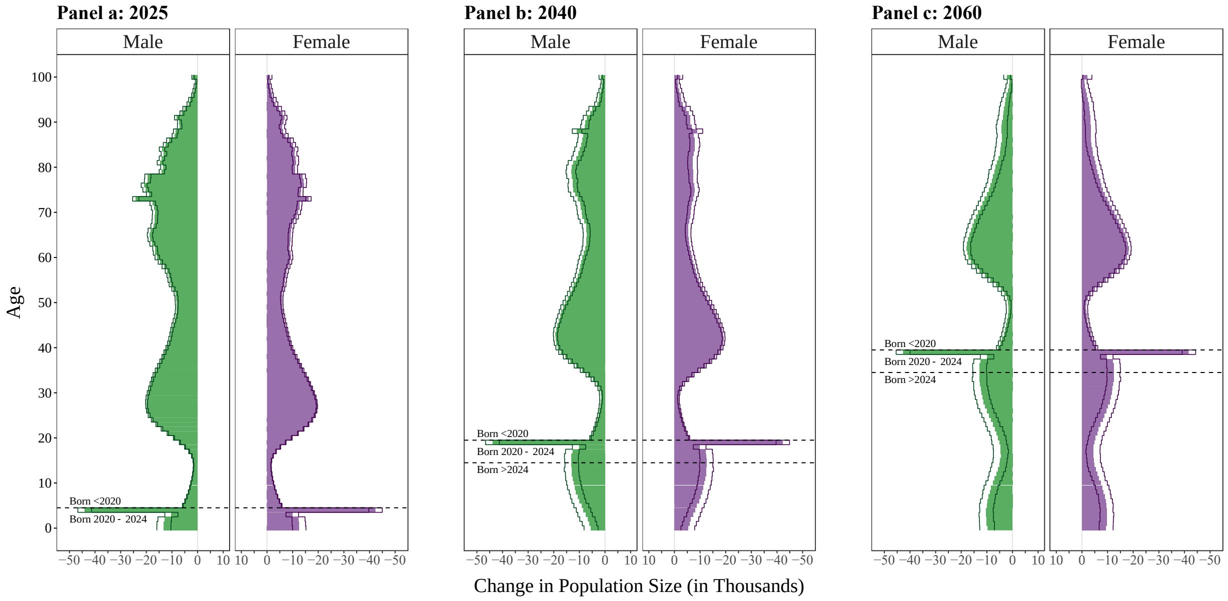

Dashed lines represent 95% confidence intervals.

C. Comparison of projections from UNWPP, Census IDB, CBO

Here we provide a comparison of the existing data sources to further illustrate our motivation to use the UNWPP. There are several organizations that produce population projections. These organizations differ in their methods, sources, and subsequently their projections. In the table below, we compile information about three different organizations and their projections: the United Nations World Population Prospects (UNWPP), the United States Census Bureau International Database (IDB), and the United States Congressional Budget Office (CBO). Overall, the UNWPP is the most transparent source with methodologically rigorous techniques. We have not identified any other organization or source with such a high data quality; hence, we argue the use of the UNWPP to address our research questions. To note, all methodological descriptions and reported projection values are for the United States. Both the UNWPP and the IDB provide projections for many (IDB) and all (UNWPP) nations. CBO provides projections for the United States alone.

The descriptions in Supplementary Table 1 are our interpretations of the methodology reports provided by each reporting agency, and any misrepresentation of their approach is our fault alone.

Supplementary Table 1. Overview of projections from the UNWPP, Census IDB, and CBO

	**UNWPP**	**IDB**	**CBO**
Publishing Institution	United Nations	U.S. Census Bureau	U.S. Congressional Budget Office
Data Link	https://population.un.org/wpp/	https://www.census.gov/programs-surveys/international-programs/about/idb.html	https://www.cbo.gov/system/files/2023-01/58612-Demographic-Outlook.pdf
Methodology Report	https://population.un.org/wpp/Publications/Files/WPP2022_ Methodology.pdf; and https://population.un.org/wpp/Download/Files/4_ Metadata/WPP2022_Data_Sources.pdf	https://www2.census.gov/programs-surveys/international-programs/technical-documentation/methodology/idb-methodology.pdf	None available
Primary Projection Tools	Bayesian hierarchical models; Cohort component projection method	Logistic curves; Cohort component projection method	Unstated
Most Recent Year of Input Data (as of June 2023)	2021	2020	2022
Projection Years	2022-2100	2021-2060	2023-2053 (though, underlying Excel documents show data until 2096)
Brief Mortality Explanation (US-specific)	Sex- and age- specific mortality rates were projected using a modified Lee-Carter method, constrained to the projected life expectancy at birth.	Logistic curve fitted to recent estimates of life expectancy at birth, by sex; age-specific mortality rates determined via “iterative interpolation”.	Not explained
Brief Fertility Explanation (US-specific)	Bayesian hierarchical model to project TFR, then to find ASFR project age-pattern of fertility forward, assuming convergence to global model age pattern of fertility	Logistic curve to determine TFR, then ASFR determined via “iterative interpolation”.	Not explained
Brief Migration	Project aggregate net migration: pre-COVID levels of net migration were kept constant until end of the century, with some adjustments made based on return migration of	Based on the composition of migrants. For *economic migrants:* Future migration is assumed to reach	Not explained

Explanation (US-specific)	refugees and government stance on migration. Then apply the model age pattern of migration (from Rogers and Castro 1981).	a level equal to the average of net economic migrants for the past 20 years or longer. For *humanitarian migrants*: future migration is assumed to reach zero.	
Data Source(s): Population	(a) 1950, 1960, 1970, 1980, 1990, 2000, 2010, 2020 Censuses (adjusted for under/over count);<br><br>(b) International estimates used up to 2021; and with estimates of the subsequent trends in fertility, mortality and international migration;<br><br>In addition, the (a) adjusted for under/over count; (b) adjusted for age heaping; (c) adjusted for under enumeration of children under age 15; (d) 2005, 2015 ACS survey estimates; (e) official estimates through 2021.	Census, 2010	Congressional Budget Office
Data Source(s): Mortality	(a) Official Estimates (US Life Tables) through 2017;<br><br>(b) registered deaths (Vital Registration) by age and sex available through 2021;<br><br>(c) International estimates used up to 2021.	Vital Registration, Preliminary 2018	Congressional Budget Office
Data Source(s): Fertility	(a) official estimates (Vital Registration) of age-specific fertility rates through 2021;<br><br>(b) registered births classified by age of mother and the underlying female population by age through 2019 (adjusted for under registration);<br><br>(c) International estimates used up to 2020;<br><br>In addition, the (a) indirect estimates obtained from the application of the reverse survival method to the 1960, 1970, 1980, 1990, 2000, 2010 censuses and 1990-2010	Vital Registration, Preliminary 2018	Congressional Budget Office; Social Security Administration

	Education Stats and 2005, 2015 ACS; (b) cohort-completed fertility backdated by the mean age of childbearing from the 1950, 1960, 1970, 1980, 1990 censuses and 1952, 1954, 1957, 1959 Survey and 1994 CPS have been considered.		
Data Source(s): Migration	(a) official figures of net international migration flows, and assumed subsequent trends in international migration;<br><br>(b) estimates of migrant flows, and assumed subsequent trends in international migration;<br><br>(c) information on foreign-born populations from censuses and registers from major countries of destination;<br><br>(d) estimates derived as the differences between overall population growth and natural increase;<br><br>(e) UNHCR statistics on the number of refugees in the main countries of asylum.	"Survey estimates used along with administrative data" (American Community Survey), 2018	Congressional Budget Office; Social Security Administration
e_0 Comparison	Projected:<br>2022 = 78.2<br>2030 = 80.8<br>2040 = 82.3<br>2050 = 83.7	Projected:<br>2021 = 80.4<br>2030 = 81.8<br>2040 = 83.2<br>2050 = 84.5	Projected:<br>2023 = 77.8<br>2030 = 79.3<br>2040 = 80.8<br>2050 = 82.0
TFR Comparison	Projected:<br>2022 = 1.66<br>2030 = 1.68<br>2040 = 1.68<br>2050 = 1.70	Projected to be 1.84 in every year.	Projected:<br>2023: 1.67<br>2030, 2040, 2050: 1.75
Net Migration Comparison (Rate per 1,000)	Projected:<br>2022 = 3.0<br>2030 = 3.0<br>2040 = 2.8<br>2050 = 2.7	Projected:<br>2020-2039 = 3.0<br>2040-2050 = 2.9	Projected:<br>2023 = 4.1<br>2030 = 3.1<br>2040 = 3.1<br>2050 = 3.0

D. All manuscript graphs, ending at 2040

We then replicate all figures to end at 2040. Notably, Figure 3 in the main manuscript already is for 2040, so is not replicated here.

Supplementary Figure 7. Absolute (Panel A) and Relative (Panel B) Differences in Population Size by Age Group and Sex between Baseline (with COVID-19) and Counterfactual (without COVID-19), 2020-2040.

Panel A

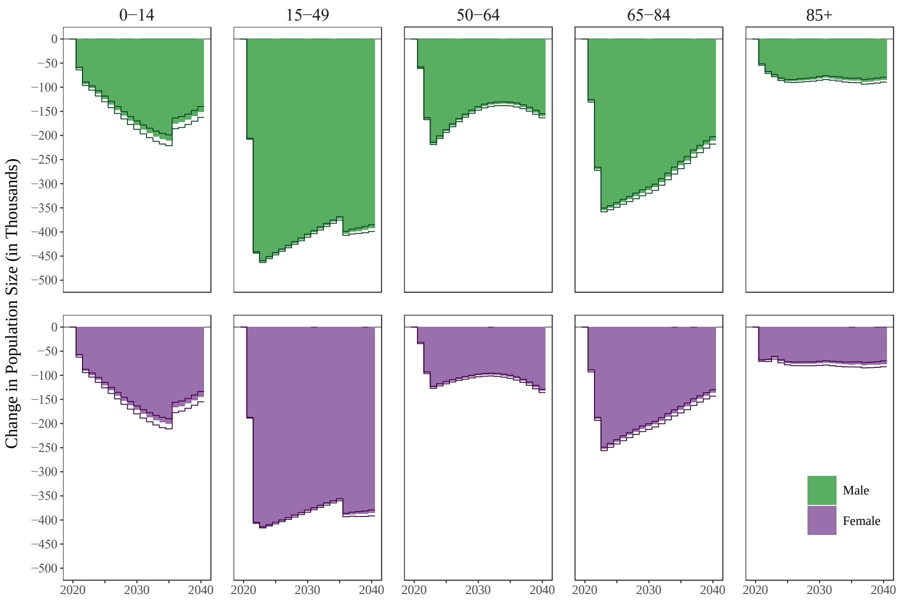

Panel B

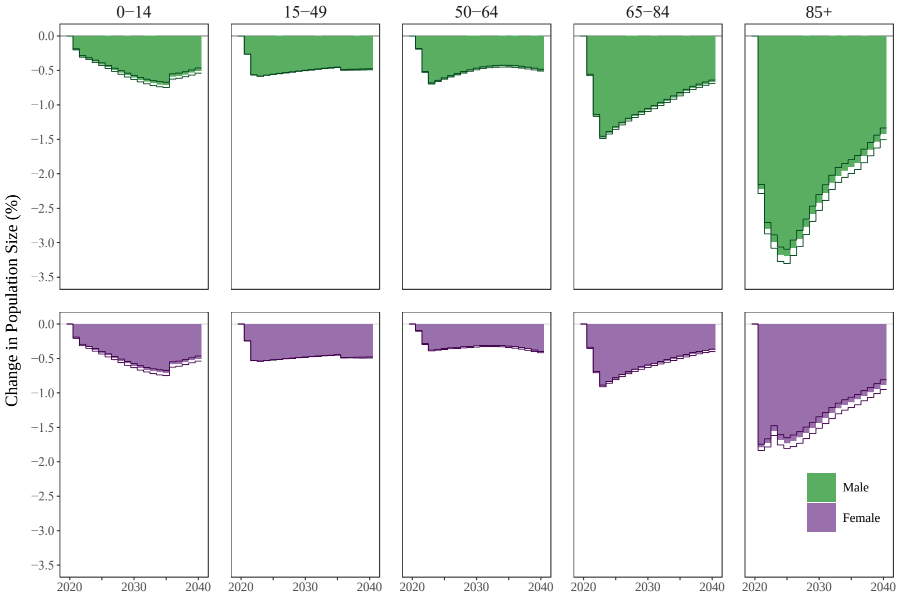

Notes. Data come from the United Nations World Population Projections and the authors' own estimates. Dashed lines represent 95% confidence intervals.

Supplementary Figure 8. Absolute (Top) and Relative (Bottom) Difference in Population Size by Age and Sex for Years 2025 (Panel A), 2035 (Panel B), 2040 (Panel C).

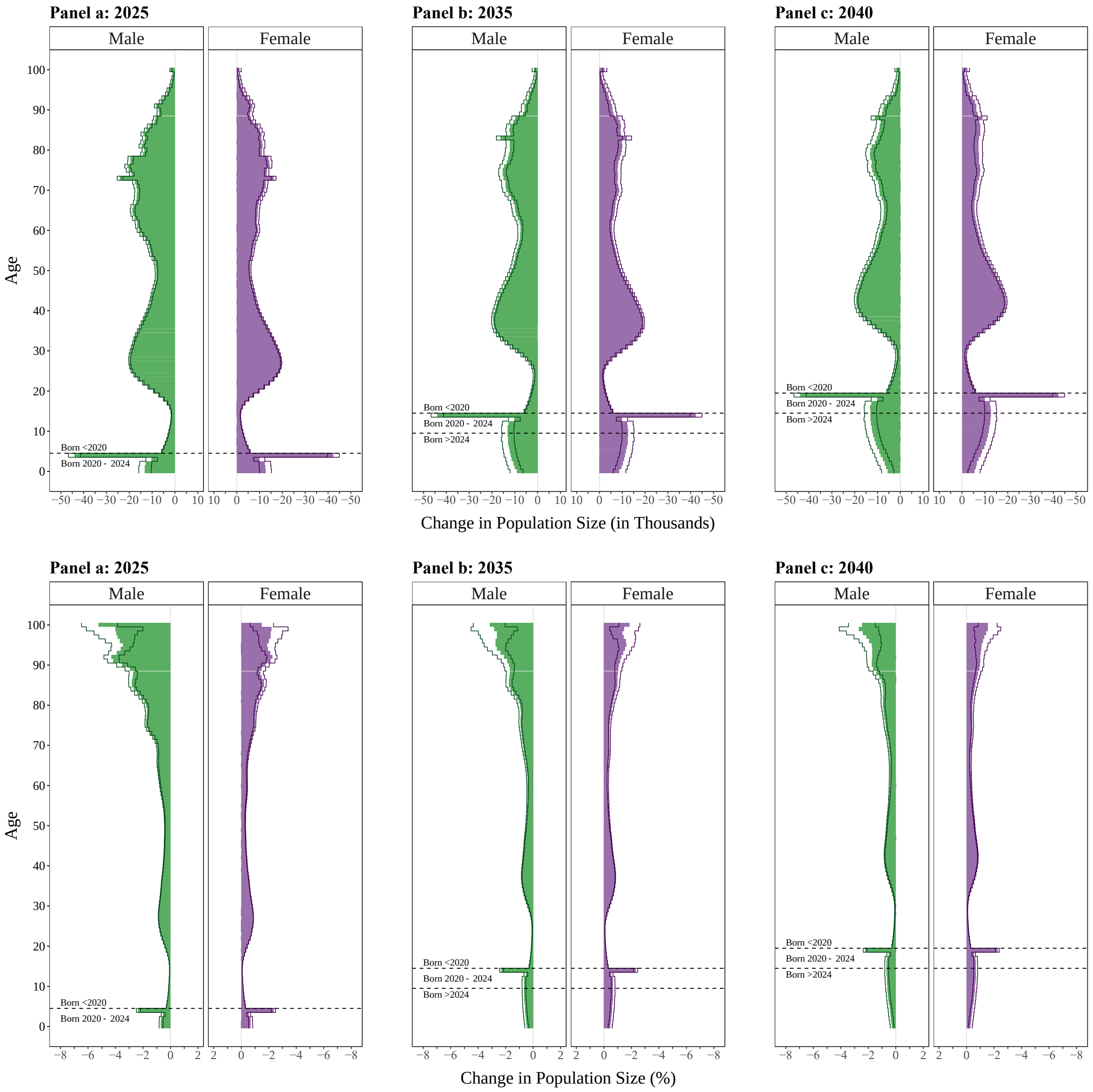

Notes. Data come from the United Nations World Population Projections and the authors' own estimates. Dashed lines represent 95% confidence intervals.

Supplementary Figure 9. Change in Dependency Ratios for Young-age (Panel A, top), Old-age (Panel B, middle), and Total (Panel C, bottom).

	Change in Dependency Ratio			
	2025	2035	2040	Trend
**Panel A: Young-age**				
All	0.03	−0.07	−0.01	
Mortality	0.04	0.01	0.01	
Fertility	−0.03	−0.03	0.01	
Migration	0.02	−0.05	−0.02	
**Panel B: Old-age**				
All	−0.19	−0.12	−0.05	
Mortality	−0.28	−0.22	−0.18	-0.28
Fertility	0.00	0.00	0.01	
Migration	0.09	0.11	0.11	
**Panel C: Total**				
All	−0.16	−0.19	−0.06	
Mortality	−0.24	−0.21	−0.17	
Fertility	−0.03	−0.03	0.02	
Migration	0.11	0.06	0.09	0.13

Notes. Data come from the United Nations World Population Projections and the authors' own estimates. Dependency ratios are calculated as the percentage of non-working population (<15 and >64) to working-aged persons for each component separately and in total. Trendlines show 2020-2040, with dots at years 2025, 2035, 2040. Dashed line is equivalent to no change. Young-Age = <15 / (15–64); Old-Age = ≥65 / (15–64); Total = (<15 + ≥65) / (15–64). Estimates for "mortality" indicate the projected difference in dependency ratios if only mortality had not changed during the pandemic, "fertility" if only fertility had not changed, and "migration" if only migration had not changed.

Supplementary Figure 10. Change in Population Share by Age Group

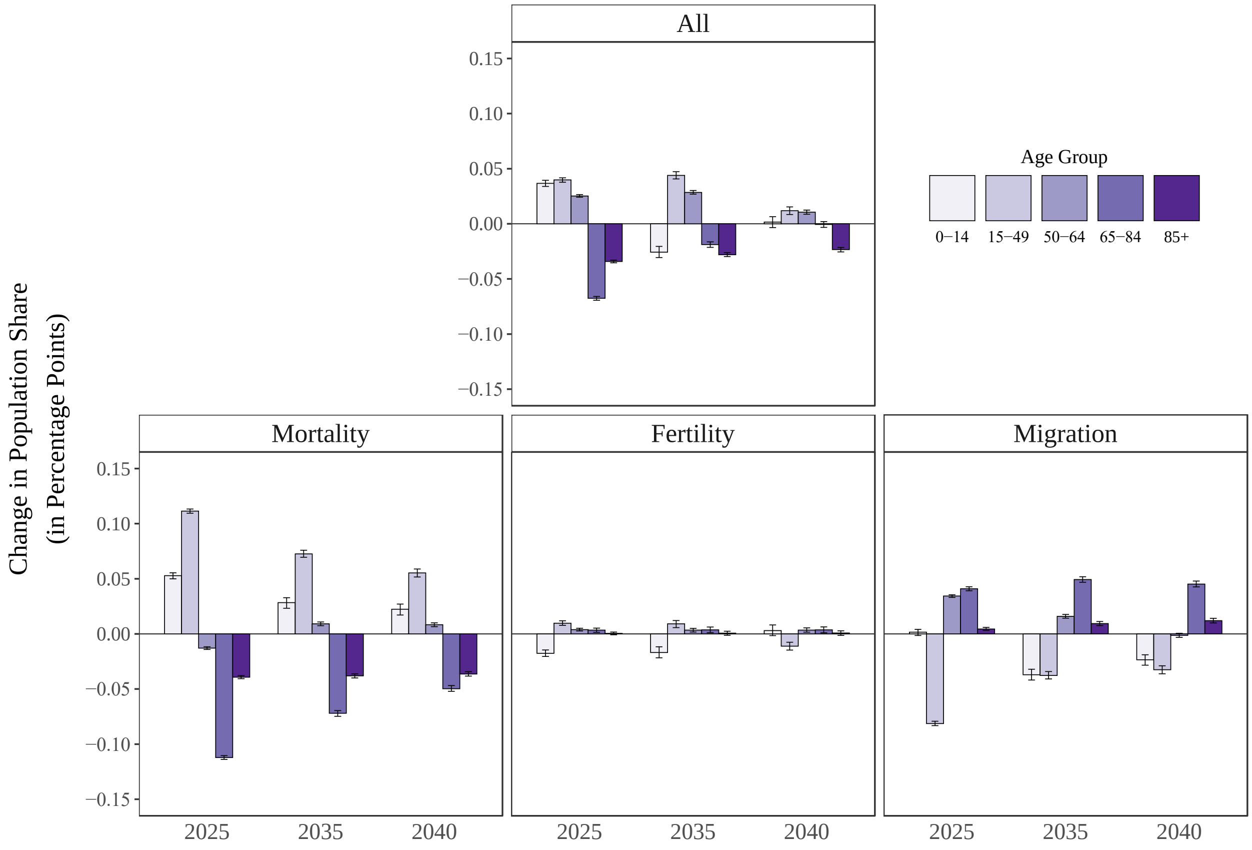

Notes. Data come from the United Nations World Population Projections and the authors' own estimates. Whiskers represent 95% confidence intervals.

E. Projected change in population size and summary table

Supplementary Figure 11. Projected Absolute (Panel A) and Relative (Panel B) change in Population Size

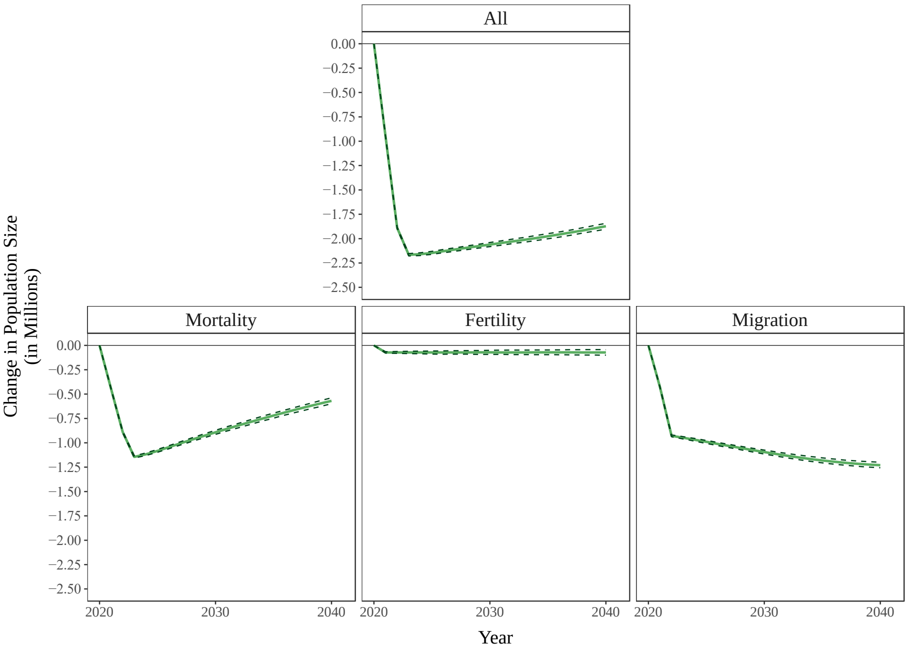

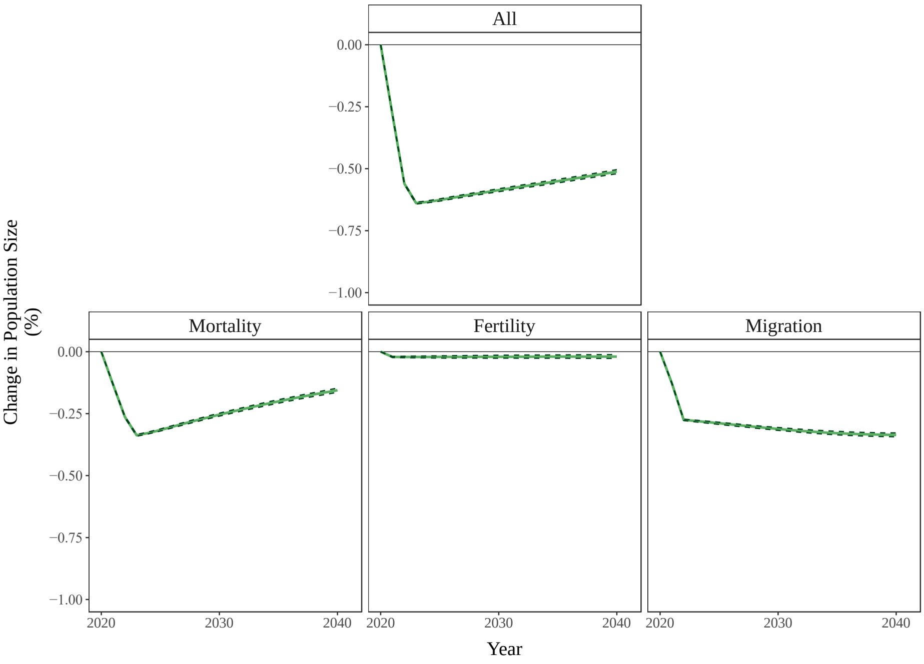

Notes. Data come from the United Nations World Population Projections and the authors' own estimates. Dashed lines represent 95% confidence intervals.

Supplementary Table 2. Summary Statistics of Model Output, for Joint Scenario

Year	Age	Population (Baseline)	Population (Baseline), lower	Population (Baseline), upper	Population (Counterfactual)	Population (Counterfactual), lower	Population (Counterfactual), upper	Difference (Absolute)	Difference (Absolute), lower	Difference (Absolute), upper	Difference (Percent)	Difference (Percent), lower	Difference (Percent), upper
2025	Total	342,691	342,680	342,702	344,837	344,827	344,848	-2,146	-2,161	-2,130	-0.63	-0.63	-0.62
2025	0-14	59,302	59,294	59,310	59,546	59,538	59,555	-244	-256	-233	-0.41	-0.43	-0.39
2025	15-49	157,837	157,835	157,838	158,688	158,686	158,690	-851	-854	-848	-0.54	-0.54	-0.54
2025	50-64	62,795	62,792	62,797	63,101	63,098	63,103	-306	-309	-302	-0.49	-0.49	-0.48
2025	65-84	55,693	55,688	55,698	56,275	56,270	56,280	-581	-588	-575	-1.04	-1.06	-1.03
2025	85+	7,063	7,060	7,066	7,225	7,222	7,228	-162	-166	-157	-2.30	-2.35	-2.23
2040	Total	365,852	365,832	365,873	367,725	367,704	367,746	-1,872	-1,903	-1,842	-0.51	-0.52	-0.50
2040	0-14	58,906	58,891	58,920	59,202	59,187	59,217	-295	-317	-273	-0.50	-0.54	-0.46
2040	15-49	160,433	160,425	160,442	161,211	161,202	161,219	-777	-789	-765	-0.48	-0.49	-0.48
2040	50-64	64,555	64,551	64,559	64,847	64,843	64,851	-291	-297	-286	-0.45	-0.46	-0.44
2040	65-84	67,344	67,336	67,351	67,691	67,684	67,697	-346	-357	-337	-0.52	-0.53	-0.50
2040	85+	14,612	14,607	14,618	14,773	14,767	14,778	-160	-168	-153	-1.10	-1.15	-1.05
2060	Total	380,134	380,101	380,166	381,790	381,758	381,823	-1,655	-1,703	-1,608	-0.44	-0.45	-0.42
2060	0-14	56,424	56,408	56,441	56,674	56,658	56,691	-249	-272	-224	-0.44	-0.48	-0.40
2060	15-49	154,802	154,782	154,820	155,380	155,361	155,400	-578	-609	-551	-0.37	-0.39	-0.36
2060	50-64	72,095	72,091	72,099	72,439	72,435	72,443	-343	-349	-337	-0.48	-0.48	-0.47
2060	65-84	75,135	75,127	75,143	75,527	75,520	75,535	-392	-403	-381	-0.52	-0.54	-0.51
2060	85+	21,676	21,670	21,684	21,768	21,761	21,774	-91	-101	-81	-0.42	-0.47	-0.38

Notes. Data come from the United Nations World Population Projections and the authors' own estimates from the complete model.

F. Cohort component projection method

The cohort component projection method is a well-documented approach for projecting the size of the and structure of a given population into the future (see, Preston et al. 2001). We use the two-sex model, meaning we account for changes to both males and females.

We conduct our projections one interval at a time using one-year time (2020, 2021, ..., 2059, 2060) and age (0, 1, ..., 100+) intervals. This means that the projected population on 1st January 2021 forms the baseline for the projection until 1st January 2022, and so forth. Our projection consists of three steps:

1) for each sex and age-group, estimating the population still alive at the beginning of the next interval, accounting for net migration flows;
2) for each age-group, computing the number of live births over the time interval;
3) using the sex-ratio at birth to determine the proportion of the born children that are males and the proportion that are females.

Step 1 of the projection, estimating the population alive at the beginning of the next interval, is calculated with the following formula (for each age, sex group):

$${}_1N_x(t+1) = {}_1N_{x-1}(t) \times \frac{{}_1L_x}{{}_1L_{x-1}} + {}_1I\,{}_x[t, t+1] \ ,$$

Where ${}_1N_x(t+1)$ is the population aged *x* to *x+1* at the end of the projection interval *t* to *t+1*, ${}_1N_{x-1}(t)$ is the population aged *x–1* to *x* at the beginning of the projection interval *t* to *t+1*, and ${}_1I_x[t, t+1]$ is the number of net migrants to age group *x* to *x+1* during the interval *t* to *t+1*. All net migrants are assumed to enter the population at the end of the projection interval, the same assumption made by UNWPP. Mortality is incorporated into the projection model via the survivorship ratio, $\frac{{}_1L_x}{{}_1L_{x-1}}$, or the proportion of people aged *x-1* to *x* still alive after one year in a stationary population subject to the life table derived from a set of age-specific mortality rates, ${}_1m_x$.

Step 2 of the projection, estimating the number of live births to women aged *x* to *x+1* in the time interval *t* to *t+1* is calculated with the following equation:

$${}_1B_x\ [t, t+1] = \ {}_1F_x\ \times \left(\frac{{}_1N_x\,(t) + {}_1N_x\,(t+1)}{2}\right),$$

where ${}_1F_x$ is the age-specific fertility rate in the age interval *x* to *x+1*. The fraction on the right-hand side approximates female person-years lived in the interval *t* to *t+1* as the average of women aged *x* to *x+1* alive at the beginning and at the end of the period *t* to *t+1*, excluding net migrants during the period *t* to *t+1* and under the assumptions of mortality described above.

Step 3 of the projection simply applies the sex-ratio at birth to ${}_1B_x\ [t, t+1]$ to obtain the number of new males and new females born in a given year. Additionally, the survivorship ratio is applied to the calculated number of live births, in order to obtain a count of male and female infants that survive until the end of the year. The process is then repeated for each year between 2020 and 2060.

G. Data Sources for Supplementary Analyses

All data used in supplementary analyses are publicly available and come from the Congressional Budget Office, the United States Census Bureau International Database, the Human Mortality Database, the Human Fertility Database, and the National Vital Statistics Systems. We provide links to access these data here.

1. Congressional Budget Office
    a. https://www.cbo.gov/system/files/2023-01/58612-Demographic-Outlook.pdf

2. United States Census Bureau International Database
    a. https://www.census.gov/programs-surveys/international-programs/about/idb.html

3. Human Mortality Database
    a. https://mortality.org

4. Human Fertility Database
    a. https://www.humanfertility.org

5. National Vital Statistics Systems data
    a. Fertility
        i. https://dx.doi.org/10.15620/cdc:122047
    b. Mortality
        i. https://dx.doi.org/10.15620/cdc:113096
        ii. https://dx.doi.org/10.15620/cdc:118271

References

Preston, Samuel H., Patrick Heuveline, and Michel Guillot. 2001. *Demography: Measuring and Modeling Population Processes*. Malden, MA: Blackwell Publishers.
