# Supplementary material for: Projecting the long-term effects of the COVID-19 pandemic on U.S. population structure
Source: Nat Commun. 2024 Mar 18;15:2409. doi: 10.1038/s41467-024-46582-4 (PMC10948855; doi:10.1038/s41467-024-46582-4)
Supplement: Supplementary file 3 — Reporting Summary [file 41467_2024_46582_MOESM3_ESM.pdf]

Corresponding author(s): Dr. Andrea M. Tilstra

Last updated by author(s): Jan 15, 2024

Reporting Summary

Nature Portfolio wishes to improve the reproducibility of the work that we publish. This form provides structure for consistency and transparency in reporting. For further information on Nature Portfolio policies, see our Editorial Policies and the Editorial Policy Checklist.

Statistics

For all statistical analyses, confirm that the following items are present in the figure legend, table legend, main text, or Methods section.

n/a	Confirmed	
☐	☒	The exact sample size (*n*) for each experimental group/condition, given as a discrete number and unit of measurement
☒	☐	A statement on whether measurements were taken from distinct samples or whether the same sample was measured repeatedly
☒	☐	The statistical test(s) used AND whether they are one- or two-sided<br>*Only common tests should be described solely by name; describe more complex techniques in the Methods section.*
☐	☒	A description of all covariates tested
☐	☒	A description of any assumptions or corrections, such as tests of normality and adjustment for multiple comparisons
☐	☒	A full description of the statistical parameters including central tendency (e.g. means) or other basic estimates (e.g. regression coefficient) AND variation (e.g. standard deviation) or associated estimates of uncertainty (e.g. confidence intervals)
☒	☐	For null hypothesis testing, the test statistic (e.g. *F*, *t*, *r*) with confidence intervals, effect sizes, degrees of freedom and *P* value noted<br>*Give P values as exact values whenever suitable.*
☒	☐	For Bayesian analysis, information on the choice of priors and Markov chain Monte Carlo settings
☒	☐	For hierarchical and complex designs, identification of the appropriate level for tests and full reporting of outcomes
☒	☐	Estimates of effect sizes (e.g. Cohen's *d*, Pearson's *r*), indicating how they were calculated

Our web collection on statistics for biologists contains articles on many of the points above.

Software and code

Policy information about availability of computer code

Data collection	No software was used for data collection.
Data analysis	Data were analysed in R Version 4.2.2. Full replication scripts can be found at our OSF repository: https://doi.org/10.17605/osf.io/te592.

For manuscripts utilizing custom algorithms or software that are central to the research but not yet described in published literature, software must be made available to editors and reviewers. We strongly encourage code deposition in a community repository (e.g. GitHub). See the Nature Portfolio guidelines for submitting code & software for further information.

Data

Policy information about availability of data

All manuscripts must include a data availability statement. This statement should provide the following information, where applicable:

- Accession codes, unique identifiers, or web links for publicly available datasets
- A description of any restrictions on data availability
- For clinical datasets or third party data, please ensure that the statement adheres to our policy

The United Nations World Population Prospects (UNWPP) 2022 data used in this study are available, under a Creative Commons license BY3.0 IGO. For ease of replication, we have created a project dataset, available in our OSF repository (https://doi.org/10.17605/osf.io/te592). This project dataset contains just the measures used in our analyses. This will allow any reader or user to replicate our analyses exactly. Data used in the supplementary index come from the

Congressional Budget Office, the United States Census Bureau International Database, the Human Mortality Database, the Human Fertility Database, and the National Vital Statistics Systems data.

Research involving human participants, their data, or biological material

Policy information about studies with human participants or human data. See also policy information about sex, gender (identity/presentation), and sexual orientation and race, ethnicity and racism.

Reporting on sex and gender	Analyses are sex-specific. This sex of individuals is available from the raw data files from UNWPP.
Reporting on race, ethnicity, or other socially relevant groupings	Data are not disaggregated by race/ethnicity or other social groupings.
Population characteristics	See below (Behavioural and Social Sciences Study Design)
Recruitment	N/A
Ethics oversight	The data used in this study are collected by an external source, fully anonymized and cannot be traced back to the individual from whom it came, thus exempt from ethics review by the University of Oxford Medical Sciences Interdivisional Research Ethics Committee.

Note that full information on the approval of the study protocol must also be provided in the manuscript.

Field-specific reporting

Please select the one below that is the best fit for your research. If you are not sure, read the appropriate sections before making your selection.

☐ Life sciences ☒ Behavioural & social sciences ☐ Ecological, evolutionary & environmental sciences

For a reference copy of the document with all sections, see nature.com/documents/nr-reporting-summary-flat.pdf

Behavioural & social sciences study design

All studies must disclose on these points even when the disclosure is negative.

Study description	Data are quantitative, and use observed and projected data available from the United Nations.
Research sample	We use four measures in our model: mortality rates and migration counts (both by year, age, and sex), female fertility rates (by year and sex) and sex ratios at birth (by year). They represent the United States population (i.e., they are not a sample but rather population counts and projections) for years 2020-2060.
Sampling strategy	The data are collected and projected by the United Nations, Population Division of the Department of Economic and Social Affairs. For the United States (the focus of the present analyses), the UNWPP collects data from: US Census, American Community Survey, Current Population Survey, Education Statistics, Population Register, and Vital Registration. These data are then compiled, and the UNWPP conducts projections. Full documentation on the UNWPP projections can be accessed via: https://population.un.org/wpp/Publications/Files/WPP2022_Methodology.pdf. No sample size calculation was performed because the data are population data.
Data collection	Data were downloaded from the UNWPP website via the download website (https://population.un.org/wpp/Download/Standard/MostUsed/). The resultant CSV files were then merged and trimmed to contain just the United States.
Timing	Data were downloaded in Fall 2022. The baseline data include years 1950-2022 and projection data cover the years 2022-2100. We limit our projection years to 2060. The 2022 version of the WPP data is the twenty-seventh edition of the data. There are not major gaps in collection periods.
Data exclusions	Only the United States was included in the present analyses. From WPP, data are available on 237 countries or areas. This paper is explicitly concerned with the United States, so all other areas were excluded.
Non-participation	No participants were involved in the study.
Randomization	Randomization is not applicable to this study because it has population level data.

Reporting for specific materials, systems and methods

We require information from authors about some types of materials, experimental systems and methods used in many studies. Here, indicate whether each material, system or method listed is relevant to your study. If you are not sure if a list item applies to your research, read the appropriate section before selecting a response.

Materials & experimental systems

n/a	Involved in the study
☒	☐ Antibodies
☒	☐ Eukaryotic cell lines
☒	☐ Palaeontology and archaeology
☒	☐ Animals and other organisms
☒	☐ Clinical data
☒	☐ Dual use research of concern
☒	☐ Plants

Methods

n/a	Involved in the study
☒	☐ ChIP-seq
☒	☐ Flow cytometry
☒	☐ MRI-based neuroimaging

Plants

Seed stocks	N/A
Novel plant genotypes	N/A
Authentication	N/A
